# Evaluation of NO Synthase Activity in Meat-Brining Solutions: Implications for Meat Curing and Color Stability

**DOI:** 10.3390/molecules30061215

**Published:** 2025-03-08

**Authors:** Marzena Zając, Rafał Szram

**Affiliations:** Department of Animal Product Technology, Faculty of Food Technology, University of Agriculture in Krakow, Balicka 122, 30-149 Kraków, Poland; rafal.szram@urk.edu.pl

**Keywords:** meat curing, arginine, color, nitrite

## Abstract

L-arginine is a substrate for nitric oxide synthase, which, in its optimal conditions in a living organism, generates nitric oxide. In this presented research, we test the hypothesis that nitric oxide can be produced in a solution in which L-arginine, inducible nitric oxide synthase, and meat are present. We evaluate the effect of L-arginine concentration (0.0%/0.1%/0.2%), temperature (20/37 °C), and incubation time (1 h/2 h) on meat color. Nitrite, L-arginine, and citrulline concentrations are analyzed, as well as the UV-Vis and Raman spectra of meat extracts and meat, respectively. The results indicate that there is very weak evidence that at a pH level closer to the enzyme’s optimum, slightly higher concentrations of nitrite can be found. The decrease in L-arginine concentration after incubation of an enzyme with meat in water suggests enzyme activity. The UV-Vis and Raman spectra do not support the generation of nitroso myoglobin. Meat color analysis showed lower a* coordinate values in samples incubated with nitric oxide synthase compared to their analogs without the enzyme. The results indicate that in given conditions, nitric oxide synthase cannot be used as a nitrite replacer.

## 1. Introduction

The use of nitrites and nitrates as meat additives has been a subject of controversy for an extended period of time. Primarily employed for preservation, they play a crucial role in inhibiting *Clostridium botulinum*. They additionally contribute to antioxidant properties, flavor enhancement, and development of the characteristic red color in meat after cooking. However, debate surrounds the presence of residual nitrite, which can potentially react with secondary amines, forming carcinogenic nitrosamines [1]. Consequently, various alternative methods are currently being proposed to mitigate residual nitrite levels and enhance the safety of meat products. Due to various nitrate functions, it is difficult to find its perfect replacement, and the proposed alternatives have their limitations. The most popular substitutes are plant powders or extracts rich in nitrates (celery and other leafy vegetables, beetroot, radishes, etc.) along with nitrate-reducing bacteria to obtain a reactive form—nitrite. Another way is to add pre-converted plant extracts, which contain nitrites. These methods, sometimes called “natural”, are considered better compared to those with traditional nitrite-curing, although the chemical substance used is exactly the same [1,2]. Data on using polyphenol-rich extracts can be found in the literature on the subject. The majority of the studies are concentrated on the inhibition of oxidation and microbial growth. As many plant constituents have the ability to enhance the conversion of nitrite to nitric oxide, meat products with these plants contain less residual nitrite. However, it is difficult to obtain the pink color effect as well as *Clostridium botulinum* inhibition [3,4].

The use of arginine as a substance to improve the color of meat has been proposed by various researchers [5,6,7]. The suggested mechanism is based on L-arginine’s ability to activate NO- synthase present in meat to generate nitric oxide, which can further react with myoglobin and other meat constituents. Bacterial strains with the ability to generate nitric oxide via L-arginine-nitric oxide synthase pathways have been identified as promising color-forming agents in meat products. The mechanisms are still not fully recognized; however, it is strongly suggested that using microorganisms is a true future alternative for nitrites [8,9,10]. Therefore, it seems valid to test and show the results of nitric oxide synthases applied to meat.

In our previous research, we used NO synthase (NOS) along with arginine to provide the maximum possible substrate concentration (9 g/100 g of meat). It turned out that although spectra analyses indicated some NO-pigment formation, no distinct visible effect, such as red color formation, was noticed [11]. We speculated that the abundance of arginine may have blocked the enzyme activity, as L-arginine may also serve as a NOS inhibitor. According to Lorin et al. [12], constitutive amounts of L-arginine in endothelial cells limit NO synthesis, which could have been a problem faced in our earlier study. Therefore, this time, we tried to create conditions for producing NO outside the meat sample—in a solution and with a much lower L-arginine concentration (0.1% and 0.2%). NO is very reactive and can quickly form peroxynitrite, oxidized NO-derived species, and react with proteins, lipids, and DNA [13]. We hypothesized that nitric oxide generated by NO synthase would be assimilated into the meat sample, giving rise to the formation of nitroso myoglobin. The aim of this study was to monitor alterations in solutions comprising NO synthase, arginine, as well as meat at various temperatures and L-arginine concentrations. The objective was to establish optimal conditions for a suitable solution for meat injection. Furthermore, two incubation temperatures were tested: 37 °C, the optimum for NO synthase activity and 20 °C, which, if effective, would provide a safer environment in terms of microbial growth and align more closely with the technologically recommended conditions.

## 2. Results and Discussion

### 2.1. pH and Color Analysis

According to the producer’s specifications (Merck, Darmstad, Germany), one unit of iNOS will produce 1.0 μmol of nitric oxide per minute at 37 °C in 50 mM HEPES, pH 7.4, containing 1 mM arginine. Two solutions, HEPES buffer (pH 7.3) and water (pH 7.5) were used to investigate the effect of lower pH on iNOS activity pH. Gonzales Arbelaez et al. [14] observed higher nitrite levels generated by nitric oxide synthase in acidic conditions (pH 6.4). Bellocq et al. [15] also reported that lower pH (7.0) upregulated iNOS activity, leading to an increase in nitrite accumulation. Based on these findings, we felt encouraged to test lower pH, and we expected it could be valid for the meat environment. Additionally, we considered the possibility of pH decrease due to L-arginine breakdown over extended incubation durations if iNOS was active. The results of pH measurements are presented in Table 1a,b. Interestingly, we observed that the introduction of meat into the HEPES solutions had no effect on pH in contrast to water samples, in which, regardless of the L-arginine concentration, the pH significantly dropped. This observation confirms the ability of the HEPES buffer to stabilize the solutions, which, in the pH conditions for enzyme activity, was very positive. According to the safety data sheet for L-arginine supplied by the manufacturer (Sigma-Aldrich, St. Louis, MI, USA), the L-arginine water solution (100 g/L) has a pH of 11.4, which means that L-arginine inclusion should increase the solution’s pH. This did not happen in the present case. Increased L-arginine concentration changed neither the pH in the HEPES buffer solution nor in the water when the enzyme and/or meat were added. Higher pH values could be obtained either by increasing the L-arginine content or by adding basic inorganic phosphates typically used in meat processing as well as phosphate alternatives such as fungi or plant-based ingredients [16].

Most of the buffer samples had a comparable pH after adding 0.1% of L-arginine, and slightly increased after adding 0.2% of L-arginine. The water samples, on the other hand, were characterized by a regular pH increase proportionally to the L-arginine concentration.

There were significant differences in pH levels between treatments when no L-arginine was added, attributed to variations between the water and HEPES buffer. However, these differences disappeared starting with the 0.1% L-arginine concentration—no statistically significant differences were observed between treatments. It can be concluded that increasing the L-arginine concentration should be sufficient to maintain optimal pH conditions for NO synthase activity and that the HEPES buffer is not necessary. Considering a practical aspect of this experiment, this is optimistic, as using water is much simpler and cost-effective.

The effect of arginine on the pH increase of meat has also been observed by other authors [17,18,19]. In our previous research, a decrease was indicated in pH after arginine inclusion was caused by using arginine hydrochloride, which has a much lower pH compared to L-arginine. However, the enzyme added to meat containing arginine hydrochloride did not affect the pH of the samples [11]. Discussing these effects is complicated due to the fact that in the research presented here, we used NO synthase along with L-arginine in a solution (with or without meat), which, to the best of our knowledge, has not been undertaken before.

It should be indicated that a strong batch-to-batch variation was observed, especially in the group of control samples. This variation may be attributed to differences in meat quality between the muscles used for each individual batch, including myoglobin content, its redox state, pH, animal breed, age, and other elements affecting meat color [20]. We assumed that these variations would become neglectable after certain treatments, such as arginine and enzyme addition. Interestingly, we observed that these variations appeared mainly for the a* chroma parameter (Table 2) when L-arginine was added, showing a significant increase with the application of 0.2% L-arginine. The average standard error for the L* parameter in the control samples was calculated as 0.46%, 0.87%, and 1.26% for 0%, 0.1%, and 0.2% of L-arginine addition, respectively. For the a* parameter, the corresponding values were 7.8%, 6.77%, and 42.09% for 0%, 0.1%, and 0.2% of L-arginine addition, respectively, while for the b* parameter, the values were 1.80%, 1.87% and 3.46% at 0%, 0.1%, and 0.2%, respectively. Similar variations were observed when the enzyme was added; however, they were lower for parameter a*, at 17.30% and 19.87% at 0.1% and 0.2% L-arginine addition, respectively. This phenomenon is challenging to explain. It could indicate enhanced enzyme activity other than NO synthase due to L-arginine inclusion. Moreover, as indicated earlier, L-arginine has been reported to decrease protein stability [21]. Therefore, it is plausible that changes in myoglobin conformation occurred, influencing the overall color of the samples.

The buffer solution had a negative effect on the lightness of all samples, both with or without the enzyme, likely due to the pH differences. Samples in water exhibited significantly higher lightness (associated with lower pH) compared to those in the HEPES buffer.

It was interesting to notice that meat samples without the enzyme and L-arginine were characterized by higher a* values compared to meat samples incubated with L-arginine. The results, as presented in Table 2, indicate that meat incubated in 0.1% L-arginine solutions showed no significant difference in terms of a* coordinate compared to control samples with no L-arginine, regardless of incubation conditions. Interestingly, incubation in a 0.2% L-arginine solution resulted in much lower a* values of meat. In the samples incubated with the buffer, both applied incubation temperatures induced a significant decrease in the a* parameter. These observations are in contrast to what was published earlier by Bludau et al. [6], who investigated beef frankfurters with the addition of L-arginine and observed increased redness. Bludeau et al. [6] suggested that L-arginine enhances the internal color of beef frankfurters, specifically, the a* value, as both L* and b* do not change. Additionally, Ras et al. [22] suggested that *Staphylococci* are capable of producing nitric oxide synthase, which, in the meat environment, can interact with myoglobin, generating the pink cured meat color.

Our assumption was that once nitric oxide is generated due to L-arginine degradation, it would react with myoglobin. However, this might not be as simple due to several reasons. The reactions taking place in meat during typical nitrite curing involve not only those with myoglobin but also with lipids, proteins, and other constituents. Only 20% of nitrite added to meat is bound to myoglobin, and it is difficult to state which of the reactions is preferential [5]. It does not matter when the nitrite is present in excess, but in our case, nitric oxide could have been involved in reactions other than hem-binding.

Moreover, NO binds faster to oxymyoglobin than to metmyoglobin [23], which might be predominant in small portions of the meat used in this study. Therefore, the expected reaction was probably not preferential to even take place.

We explored distinctions between the control and iNOS samples with an equal level of L-arginine. A noteworthy discovery emerged: HEPES buffer samples with 0.1% of L-arginine incubated at 37 °C, as well as all the samples incubated at 20 °C, had significantly lower a* values when iNOS was added. No such difference was observed for samples incubated at 37 °C in water. Furthermore, when the L-arginine concentration was increased to 0.2%, those significant differences disappeared. It is possible that the HEPES buffer caused myoglobin to dissolve in the solution, which would be confirmed by UV-Vis spectra (more distinct Soret peak confirming myoglobin presence).

No other specific trend was observed, except for one clear tendency: the use of NO synthase in the solution not only failed to enhance color formation but also led to a decrease in the crucial a* color parameter—which significantly influences the visual impression of cured meat. It is possible that due to L-arginine’s ability to dissolve proteins [24], its increased concentration enhanced myoglobin solubility and its subsequent extraction to the solution.

### 2.2. Nitrite Content

Nitric oxide, being a highly reactive compound, is typically oxidized to nitrite. Gupta & Kaiser [25] reported that approximately 70% of NO produced by iNOS can be oxidized within the first 30 min of the reaction. Consequently, nitrite content analysis is often employed to assess NO synthase activity. In our research, the NO_2_^−^ concentration in the control solutions containing meat at three concentrations of L-arginine (0%, 0.1%, and 0.2%) without the enzyme and in solutions containing the enzyme, with or without meat at two concentrations of L-arginine (0.1 and 0.2%), is presented in Table 3. It can be observed that the NO_2_^−^ ion was detected in samples where neither L-arginine nor the enzyme was applied. Nitrites and nitrates can be found in fresh meat and not cured meat products originating from nitrogen metabolism [1,26]. Moreover, in buffer solutions with 0.1% L-arginine, the NO_2_^−^ ion concentration was significantly higher in the samples containing the enzyme. This strongly suggests that the enzyme enhanced NO production.

Current regulations concerning nitrite addition allow for a maximum of 120 mg of sodium nitrite per 1 kg of meat products [27]. This concentration is much higher than that required to obtain the cured meat color, antioxidant effect, and microbiological safety. The residual nitrite is necessary to maintain color stability and overall quality, as well as the safety of the product. However, it is worth decreasing the residual nitrite content, which can enhance carcinogenic nitrosamine production, while using other methods to maintain the safety and antioxidant effect [1]. Therefore, despite low nitrite levels in our tested samples, it could be expected that reactions involving color formation would occur.

The information on what is the minimum concentration of nitrite to fulfill all its functions is scarce. According to Wang et al. [28], 50 mg of nitrite is sufficient to develop desirable color and inhibit oxidation. Assuming that at least 50 mg per 1 kg of meat is necessary, it must be noted that the highest concentration detected in our research was approximately 0.1 mg/kg of meat, which is insufficient. On the other hand, considering the assumption that NO production would be continuous in the solution with the enzyme and that it would be continuously consumed by meat placed in the solution, the concentration measured in the solutions after incubation should be treated as the residual nitrite, thus, the part of the nitrite that is left after it had reacted with the meat constituent. Still, considering the effect of color, the NO concentration was not sufficient.

According to Zhang et al. [29], who analyzed marinated chicken breasts, L-arginine enhances the extraction of salt-soluble proteins. The authors found more salt-soluble proteins in marinades containing L-arginine. Based on that, we speculated nitrite may be extracted along with the proteins. Kato and Kikugawa [30] demonstrated that some amino acids have the ability to react with the nitrite; therefore, solutions with meat should exhibit lower nitrite concentrations, a phenomenon not confirmed in our study. No spectacular color effect caused by NO synthase added to the solutions was observed. The slightly higher concentrations of nitrite noted in samples with the enzyme are difficult to explain. Other HEPES samples demonstrated a similar trend, although it was not statistically confirmed. Thus, it is possible that samples characterized by a pH level close to the optimal enzyme pH exhibited increased nitrite production, which would confirm iNOS activity.

### 2.3. Arginine and Citrulline Content

L-arginine is the substrate for NO synthase. NO production requires hydroxylation of L-arginine to N-hydroxy-L-arginine, after which it is oxidized, resulting in citrulline and NO production [31]. Our hypothesis was that along with NO synthase activity, the content of L-arginine in the solution would decrease while the citrulline content would increase proportionally. Testing the concentration of both amino acids (arginine and citrulline) could be a method for monitoring enzyme activity.

Before conducting experiments with the NO-synthase, a separate set of analyses were run on solutions containing meat, no enzyme, and with or without added L-arginine. Clearly (Table 4), all the solutions with added L-arginine exhibited a higher content after incubation. However, it was observed that for the solution in which L-arginine was not initially present, some amounts of this compound appeared, leading to the simple conclusion that it was extracted from the meat during incubation. Pork *M. semimembranosus* (used in this study) contains 1.81 g/100 g of L-arginine [32]. The pork neck muscle analyzed by Bahelka et al. [33] varied in arginine content depending on whether it was sampled from surgical castrates (1.49%), gilts (1.59%), or entire males (1.61%). The same applies to citrulline, which in our study was not detected in the solutions lacking meat. *M. longissimus thoracis*, depending on the animal’s age, contains from 0.013 to 0.026 g/100 g of L-arginine and from 0.002 to 0.01 g/100 g of citrulline [34].

Higher levels of L-arginine were found in samples containing meat. In two cases, these amounts exceeded the added L-arginine level (100 mg/100 mL). This could be explained by additional L-arginine coming from meat. Arginine serves as a solvent additive in many biotechnological applications. It helps refold proteins and solubilize them from inclusion in bodies [21]. A similar situation was observed for citrulline, which was not detected in the solutions without meat and appeared when meat was present. In water solutions, at both incubation temperatures, a significant decrease of L-arginine could be observed. This may be proof of NO synthase activity, which we anticipated. It could be expected that the citrulline content would increase in these samples. However, we did not observe such a situation, probably due to some intermediate products that may appear in the L-arginine-citrulline cycle [35].

### 2.4. UV-Vis Analysis

The characteristic red color of meat is attributed to the presence of the pigment—myoglobin. The myoglobin levels in pork, as in other animal species, vary based on phenotype, genotype, and muscle type, ranging from 0.51 to 2.17 mg/g [36]. The UV-Vis spectra provide valuable information about the electronic transitions occurring within the heme group.

**Figure 1:**
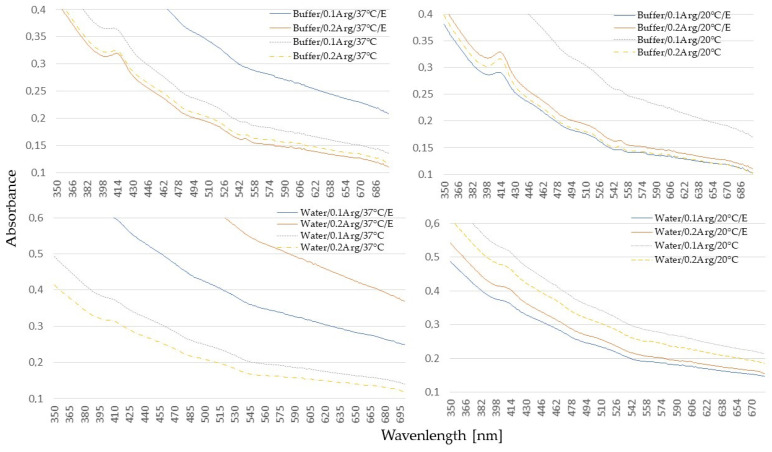
UV-VIS spectra of meat extracts after incubation in buffer/water in 0.1/0.2% concentration of L-arginine at 20 °C/37 °C with (E) or without enzyme.

The UV-Vis spectra of meat extracts containing myoglobin typically display a distinctive Soret band (UV region) between 390 and 440 nm. This band is associated with a strong electronic transition (π→π*), characteristic of all heme proteins. Additionally, weaker Q bands are located in the visible range between 500 and 700 nm and are found useful in differentiation between ferrous and ferric heme species [37]. Specifically, two bands are observed in the Q band region: one located at approximately 540–550 nm (Q_v_) and the other at 570–580 nm (Q_0_). However, as evidenced by our study, the Mb meat extracts are characterized by very weak or even the absence of these bands (Figure 1). We speculated that this could be attributed to a small sample amount (5 g), for which most of the myoglobin was lost during the extraction process, or due to a total denaturation and destruction of myoglobin and, therefore, its absence in the sample. It is noteworthy that a weak Soret band only appeared in samples with the HEPES buffer, which could protect the Mb from denaturation. In water samples, it was either absent or very weak. This observation could explain the slightly higher a* color values in the control samples incubated in the HEPES buffer (Table 2). Regarding Q-bands in the analyzed samples, some very weak signals appeared again in samples with HEPES, slightly stronger compared to water extracts.

The most intriguing aspect of this study was the information about NO-myoglobin (NOMb) presence in the tested solutions. According to Millar et al. [38], NOMb exhibits two high peaks, at 548 nm and 579 nm. Its spectrum resembles that of oxymyoglobin, however, the first peak is higher than the second one, in contrast to oxymyoglobin. The existence of these two peaks in nitrite-cured meat has been confirmed by other researchers [39,40]. Our results indicate that in some of the samples, only the first peak was discernible, albeit very weak. Furthermore, both samples with the enzyme and the control samples exhibited comparable excitation peaks in this region. Ning et al. [39] reported similarly weak peaks, but the signals became much stronger after the introduction of L-lysine or L-arginine. This finding was consistent with that obtained in our previous research [11]. Consequently, it was expected that if NOMb was present in the samples, its signal could be enhanced by the presence of L-arginine. However, none of these signals were achieved.

It is crucial to emphasize that any attempt to interpret the state of myoglobin based solely on the presented charts would be inappropriate and could potentially lead to erroneous conclusions.

### 2.5. Raman Spectra

**Figure 2:**
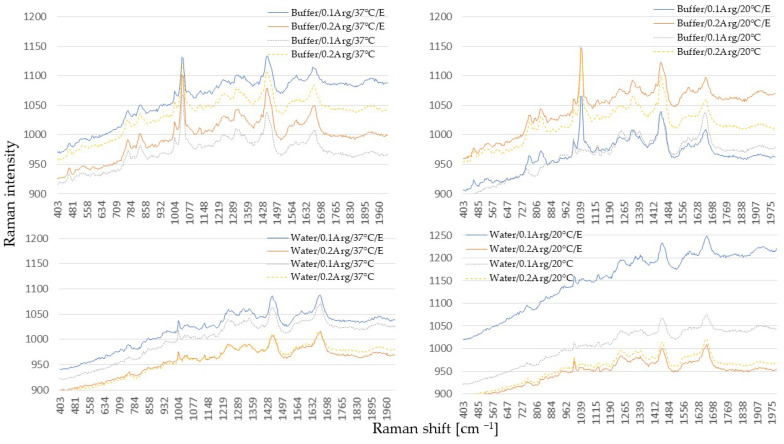
Raman spectra of meat samples after incubation in buffer/water in 0.1/0.2% concentration of L-arginine at 20 °C/37 °C with (E) or without enzyme.

Raman spectroscopy is a technique that provides information about the molecular vibrations and structural characteristics of a substance by measuring the inelastic scattering of monochromatic light [41]. In the context of myoglobin, this technique is often employed to study heme group properties, its heme iron ion oxidation state, and the presence of various ligands. Raman spectroscopy, in conjunction with other spectroscopic and analytical techniques, provides a more comprehensive understanding of the molecular composition of the heme porphyrin ring. The state of myoglobin, particularly the coordination and oxidation states of the heme iron, is elucidated through intense Raman spectra and the core-marker bands present within the 1300–1700 cm^−1^ spectral range [42]. The ν_4_ band found between 1356 and 1378 cm^−1^ is considered a marker of the heme iron ion oxidation state. In ferrous-containing samples, this band is situated between 1356 and 1360 cm^−1^, while in ferric samples, it shifts to a range between 1368 and 1378 cm^−1^ [43]. The results from our study, after subjecting meat samples to Raman spectra analysis, were compared to the data presented by Ma et al. [44,45]. It was indicated that nitrosomyoglobin presence in Raman spectra should be manifested in the redshift to approximately 1374 cm^−1^ (for oxidized or oxygen-free form). The Raman mode ν3 and ν2 should be detected at 1482 and 1564 cm^−1^, respectively. The very weak peaks within the region of 400–600 cm^−1^ noted in our study (Figure 2) could indicate the lack of myoglobin in the sample or the presence of very small, challenging-to-detect amounts of this protein, which remained in agreement with the UV-Vis data (Figure 1). This could have been due to a very small sample used in this study (5 g). In another study by Ma et al. [46], conducted on yak meat myoglobin during wet curing, the authors revealed that sodium nitrite expanded and then compressed myoglobin amide I (1340–1390 cm^−1^), α-carbon, and the atoms. The size of the middle part of the hem ring structure in amide III (1535–1575 cm^−1^) increased at first and then decreased. What is interesting is that the samples with NO synthase in our study usually exhibited higher intensity bands related to Mb presence compared to the control samples. Additionally, in Raman spectra of some samples extracted in HEPES (e.g., A0.2), a band at around 1560 cm^−1^ could be noted, and the shoulder was at 1314 cm^−1^, which may be assigned to the heme-nitrosylated complexes [47]. The formation of NOMb can also be confirmed by the presence of very weak peaks at 549 cm^−1^, which again, were observed in the spectra from HEPES-incubated samples. Spectra of meat incubated in HEPES were distinct compared to the water-extracted samples due to the presence of strong Raman band origination from SO_3_ stretching vibration at 1046–1048 cm^−1^. This finding aligns with the data presented by other authors, i.e., Xi and Haes [48,49].

## 3. Materials and Methods

### 3.1. Materials

*M. semimembranosus* pork meat (fat 2.0 g/100 g, protein 22.4 g/100 g) was purchased from a local retailer (Macro Cash and Carry, Kraków, Poland). It was ground using the MADO Primus grinder (Dornhan, Germany) with a 3-mm plate. The meat mass was mixed, before dividing it into portions, and then 5 g balls were formed. Four independent muscles were purchased for each of the four conducted experiments. Chemical reagents were supplied as follows: L-arginine, NO synthase (iNOS recombinant, expressed in *E. coli*, buffered aqueous solution), sodium nitrite, HPLC water, acetate-phosphate buffer, acetonitrile—Merck Life Science Sp. z o.o., an affiliate of Merck KGaA, Darmstadt, Germany; Standards, buffers for arginine and citrulline analysis Waters (Golden, USA); HEPES buffer (Carl Roth GmbH + Co. KG, Karlsruhe, Germany).

### 3.2. Meat Sample Preparation

L-arginine solutions (0.1% and 0.2%) were prepared in water or a HEPES buffer, and the NO-synthase enzyme (1 U) (Sigma Aldrich, St Luis, MI, USA) was added into 30 mL of all the solutions. Test samples with and without meat (5 g) were prepared. The concentrations of both L-arginine and NO synthase were chosen based on the enzyme specification, our previous experience, and preliminary studies. The solutions with and without meat were incubated at 37 °C or 20 °C (Incubator Hera Therm, ThermoScientific, Waltham, MA, USA) for 1 h or 2 h. After the incubation period, the samples were heated for 15 min in a water bath (95 °C) to inactivate the enzyme and to develop the red color of the cured meat.

The control samples with meat and L-arginine solutions (0.1 and 0.2%) without the enzyme were prepared at the same time and in analogous temperature conditions as described above.

### 3.3. pH Measurement

pH was measured in the solutions after cooking and cooling using a pH meter (Elmetron Cp-505 electrode, Zabrze, Poland). In samples for which meat was used, the pH was measured after filtration. The instrument was calibrated using standard phosphate buffers (pH 4.0 and 7.0) and corrected for temperature.

### 3.4. CIELab Color Analysis

Color analysis (CIELab system) of the cooked meat samples was conducted using the Konica Minolta colorimeter (Konica Minolta CM-3500d, Osaka, Japan) after calibrating the device according to the manufacturer’s instructions (using white and black enamel). The measurement mode was D/8 (SCE), D65 illuminate, with a 10° viewing angle. The results were represented by L* (lightness), a* (redness), and b* (yellowness) values.

### 3.5. Nitrite Content Analysis

Nitrite and nitrate content analyses were conducted in the solutions described in our previous article [50], with some modifications. For the analysis, 4 mL of the filtered solution was mixed with 4 mL of the Griess reaction solution and left for 20 min. Absorbance (λ = 520 nm) was measured using a spectrophotometer (Helios γ07-056, Thermo Scientific, Waltham, MA, USA). The concentration of nitrite was measured using the standard curve prepared beforehand. All the analyses were conducted in duplicate. The nitrite content was presented as µg/1 mL of the solution.

### 3.6. Arginine and Citrulline Content Analysis

#### 3.6.1. Sample Preparation

Arginine and citrulline concentrations were analyzed using the HPLC method in the reverse phase mode using the ACCQ Tag analytical kit from Waters (USA). A liquid sample (10 µL, straight after incubation and filtration) was mixed with 70 µL of borate buffer (pH 8.2–9.0), after which 20 µL of 6-aminoquinolyl-N-hydroxysuccinimidylcarbamate (AQC) (3 mg/mL acetonitrile) was added.

#### 3.6.2. HPLC Analysis

The Dionex Ultimate 3000 HPLC system (Thermo Scientific, Waltham, MA, USA) was utilized for separation. The Nova-Pak C18 column (4 μm, 150 mm × 3.9 mm) from Waters (Golden, USA) was employed. In the elution process, an acetate-phosphate buffer (pH 5.2) was used as eluent A and a 60:40 acetonitrile/water mixture as eluent B. The column was maintained at 37 °C, with detection parameters set to an excitation wavelength of 250 nm and an emission wavelength of 395 nm. Amino acid quantification was performed using one-point calibration with analytical standards at a concentration of 50 pM for each [51].

The results were calculated using Chromeleon 7.0 software and expressed as µg of L-arginine or citrulline in 1 mL.

### 3.7. UV-Vis Spectra Analysis of Myoglobin Forms

The extracts for myoglobin forms and UV-Vis spectra analyses were prepared as described in our previous work [11].

### 3.8. Raman Data Acquisition and Analysis

Raman analyses were conducted on cooked meat used for incubation, separated from the solution. The analyses were conducted as described in our previous work [11].

### 3.9. Statistical Analysis

The results were analyzed using Statistica v 13.0 software (Tibco, Palo Alto, CA, USA). Four independent experiments were conducted with two repetitions for each variant presented. The Shapiro-Wilk test was conducted to determine the normality of the results. ANOVA was performed where the batch was treated as a random effect, and L-arginine concentration, enzyme/no enzyme, meat/no meat, and buffer/water were considered fixed effects. The differences between individual groups were established using Dunkan’s post hoc test at *p* < 0.05. The results are presented as mean values ± standard errors.

## 4. Conclusions

The basic aim of this study was to test the conditions for NO synthase activity in solutions in which meat could be immersed and used further as a brine for meat curing; therefore, the most interesting was the effect of the meat’s color change. This effect was not observed. Moreover, we found some evidence that iNOS could decrease the a* coordinate values, indicating a red color of the meat. As some evidence of iNOS activity was noted, further tests could be conducted by applying prolonged incubation time, different types of NO synthase, or additional cofactors necessary for NO activity, which we assumed would be present in meat. Furthermore, additional trials, for example, on hot-boned meat, could be conducted to enable the use of still-functioning mechanisms of enzyme activity, naturally high pH, and cofactors present in meat before rigor mortis. We conducted a series of other analyses to establish if any NO synthase activity could be detected. Maintaining iNOS activity through arginine or citrulline concentration cannot be used as an accurate tool, especially when meat is present. Amino acids extracted from meat during the incubation interfere with the L-arginine added to the solution. Nitrite content was increased in samples with the HEPES buffer and pH close to iNOS optimal, which can suggest some weak enzyme activity. UV-Vis and Raman spectra did not show any evidence of nitroso myoglobin formation. All the obtained results indicate weak evidence that NO synthase is active, given the proposed experimental conditions. The results do not support the hypothesis that NO could be produced in a solution and further absorbed by meat myoglobin as well as other meat constituents.

## Figures and Tables

**Table 1:** (**a**) pH values of solutions after incubation with or without NO synthase (mean values ± standard errors). (**b**) pH values of solutions after incubation with NO synthase, including or without meat (mean values ± standard errors).

**(a)**				
**L-Arginine Concentration (%)**	**Incubation Temperature (** **°C)**	**Buffer/Water**	**Enzyme/No** **Enzyme**	**pH**
0%	37 °C	Buffer	Yes	
			No	7.2 ^bcd^ ± 0.0
		Water	Yes	
			No	5.9 ^g^ ± 0.1
	20 °C	Buffer	Yes	
			No	7.2 ^bcd^ ± 0.0
		Water	Yes	
			No	6.0 ^g^ ± 0.1
0.1%	37 °C	Buffer	Yes	7.4 ^bc^ ± 0.1
			No	7.2 ^bcd^ ± 0.0
		Water	Yes	7.1 ^cde^ ± 0.0
			No	6.9 ^ef^ ± 0.1
	20 °C	Buffer	Yes	7.3 ^bc^ ± 0.1
			No	7.3 ^bcd^ ± 0.0
		Water	Yes	7.0 ^def^ ± 0.1
			No	6.8 ^f^ ± 0.1
0.2%	37 °C	Buffer	Yes	7.3 ^bcd^ ± 0.0
			No	7.7 ^a^ ± 0.2
		Water	Yes	7.0 ^def^ ± 0.1
			No	7.5 ^b^ ± 0.1
	20 °C	Buffer	Yes	7.3 ^bcd^ ± 0.0
			No	7.8 ^a^ ± 0.2
		Water	Yes	7.0 ^def^ ± 0.1
			No	7.8 ^a^ ± 0.2
**(b)**				
**L-Arginine Concentration (%)**	**Incubation Temperature (** **°C)**	**Buffer/Water**	**Meat/No Meat**	**pH**
0.1%	37 °C	Buffer	Yes	7.4 ^abc^ ± 0.1
			No	7.4 ^abc^ ± 0.1
		Water	Yes	7.1 ^d^ ± 0.0
			No	7.5 ^a^ ± 0.0
	20 °C	Buffer	Yes	7.3 ^abc^ ± 0.1
			No	7.5 ^a^ ± 0.1
		Water	Yes	7.0 ^d^ ± 0.1
			No	7.4 ^abc^ ± 0.0
0.2%	37 °C	Buffer	Yes	7.3 ^c^ ± 0.0
			No	7.3 ^bc^ ± 0.0
		Water	Yes	7.0 ^d^ ± 0.1
			No	7.3 ^bc^ ± 0.0
	20°C	Buffer	Yes	7.3 ^bc^ ± 0.0
			No	7.3 ^bc^ ± 0.0
		Water	Yes	7.0 ^d^ ± 0.1
			No	7.3 ^c^ ± 0.1

^a,b,c,d,e,f,g^—different letters in superscript indicate significant differences between means in the same column at *p* < 0.05.

**Table 2:** Color parameters of meat samples with different L-arginine concentration. including or without NO synthase, incubated in water or HEPES buffer samples at the temperature of 37 °C or 20 °C (mean values ± standard errors).

L-Arginine Concentration	Incubation Temperature (°C)	Buffer/Water	Enzyme/No Enzyme	L* (D65)	a* (D65)	b* (D65)
0%	37 °C	Buffer	Yes			
			No	59.34 ^ghi^ ± 0.25	1.35 ^abc^ ± 0.06	12.61 ^abc^ ± 0.18
		Water	Yes			
			No	64.04 ^cd^ ± 0.21	1.11 ^bcde^ ± 0.05	12.88 ^ab^ ± 0.12
	20 °C	Buffer	Yes			
			No	59.04 ^i^ ± 0.23	1.18 ^abcd^ ± 0.13	12.02 ^cdef^ ± 0.26
		Water	Yes			
			No	63.40 ^d^ ± 0.15	1.41 ^ab^ ± 0.03	12.83^a b^ ± 0.09
0.1%	37 °C	Buffer	Yes	59.30 ^hi^ ± 0.20	0.65 ^f^ ± 0.16	11.49 ^fg^ ± 0.18
			No	60.02 ^fghi^ ± 0.42	1.35 ^abc^ ± 0.06	12.38 ^abcd^ ± 0.20
		Water	Yes	64.18 ^cd^ ± 0.33	1.15 ^bcde^ ± 0.07	12.77 ^ab^ ± 0.15
			No	65.08 ^bc^ ± 0.38	1.13 ^bcde^ ± 0.06	12.72 ^ab^ ± 0.15
	20 °C	Buffer	Yes	59.13 ^hi^ ± 0.22	0.66 ^f^ ± 0.10	11.30 ^g^ ± 0.18
			No	60.34 ^efgh^ ± 0.49	1.51 ^a^ ± 0.08	12.58 ^abc^ ± 0.15
		Water	Yes	64.40 ^bcd^ ± 0.27	1.02 ^cde^ ± 0.10	12.95 ^a^ ± 0.13
			No	64.63 ^bcd^ ± 0.38	1.26 ^abc^ ± 0.07	12.62 ^abc^ ± 0.14
0.2%	37 °C	Buffer	Yes	61.06 ^ef^ ± 0.50	0.90 ^def^ ± 0.18	12.60 ^abc^ ± 0.36
			No	59.36 ^ghi^ ± 0.59	0.64 ^f^ ± 0.15	11.98 ^cdef^ ± 0.30
		Water	Yes	65.60 ^ab^ ± 0.45	0.82 ^ef^ ± 0.13	12.22 ^bcde^ ± 0.24
			No	66.33 ^a^ ± 0.40	0.63 ^f^ ± 0.08	11.89 ^defg^ ± 0.15
	20 °C	Buffer	Yes	61.50 ^e^ ± 0.55	0.91 ^def^ ± 0.12	12.50 ^abcd^ ± 0.30
			No	60.57 ^efg^ ± 0.54	0.59 ^f^ ± 0.17	11.87 ^cdef^ ± 0.34
		Water	Yes	66.39 ^a^ ± 0.60	0.68 ^f^ ± 0.16	12.19 ^bcde^ ± 0.24
			No	66.59 ^a^ ± 0.44	0.57 ^f^ ± 0.08	11.70 ^efg^ ± 0.13

^a,b,c,d,e,f,g,h,i^—different letters in superscript indicate significant differences between means in the same column at *p* < 0.05.

**Table 3:** Nitrite content in solutions containing meat after incubation with or without NO synthase depending on L-arginine concentration (mean values ± standard errors).

L-Arginine Concentration (%)	Incubation Temperature (°C)	Buffer/Water	Enzyme/No Enzyme	NO_2_^−^ (µg/mL)
0%	37 °C	Buffer	No	0.48 ^abcde^ ± 0.06
		Water	No	0.51 ^abcde^ ± 0.20
	20 °C	Buffer	No	0.55 ^abc^ ± 0.06
		Water	No	0.52 ^abcd^ ± 0.10
0.1%	37 °C	Buffer	Yes	0.61 ^ab^ ± 0.06
		Water	Yes	0.24 ^efg^ ± 0.05
	20 °C	Buffer	Yes	0.68 ^a^ ± 0.08
		Water	Yes	0.40 ^abcdef^ ± 0.08
	37 °C	Buffer	No	0.27 ^defg^ ± 0.03
		Water	No	0.07 ^g^ ± 0.02
	20 °C	Buffer	No	0.35 ^bcdef^ ± 0.02
		Water	No	0.13 ^fg^ ± 0.02
0.2%	37 °C	Buffer	Yes	0.48 ^abcde^ ± 0.06
		Water	Yes	0.29 ^cdefg^ ± 0.05
	20 °C	Buffer	Yes	0.48 ^acde^ ± 0.07
		Water	Yes	0.41 ^abcde^ ± 0.08
	37 °C	Buffer	No	0.35 ^bcdef^ ± 0.10
		Water	No	0.03 ^g^ ± 0.02
	20 °C	Buffer	No	0.41 ^abcde^ ± 0.12
		Water	No	0.35 ^bcdef^ ± 0.15

^a,b,c,d,e,f,g^—different letters in superscript indicate significant differences between means in the same column for the group in which meat was present at *p* < 0.05.

**Table 4:** L-arginine and citrulline content in solutions incubated with or without meat, including/without NO synthase (mg/mL) (mean values ± standard errors).

Incubation Temperature (°C)	Buffer/Water	Meat/No Meat	Enzyme/No Enzyme	L-Arginine mg/100 mL	Citrulline
37 °C	Buffer	No	yes	68.73 ^ef^ ± 4.62	nd
		Yes	yes	88.55 ^cd^ ± 3.33	9.85 ^a^ ± 0.54
			No	95.67 ^bc^ ± 5.81	9.89 ^a^ ± 0.81
	Water	No	yes	55.85 ^f^ ± 4.47	nd
		Yes	yes	84.76 ^cd^ ± 6.00	7.67 ^ab^ ± 1.03
			no	109.73 ^ab^ ± 1.49	8.71 ^ab^ ± 0.61
20 °C	Buffer	No	yes	59.99 ^f^ ± 4.33	nd
		Yes	yes	79.26 ^de^ ± 6.62	8.48 ^ab^ ± 0.60
			no	94.75 ^bcd^ ± 4.81	7.15 ^b^ ± 0.65
	Water	No	yes	66.77 ^ef^ ± 6.21	nd
		Yes	yes	84.67 ^cd^ ± 4.59	8.86 ^ab^ ± 1.06
			no	123.28 ^a^ ± 3.01	9.59 ^ab^ ± 0.57

nd—not detected. ^a,b,c,d,e,f^—different superscript letters in the same columns indicate significant differences between samples.

## Data Availability

The original contributions presented in this study are included in the article/Supplementary Material. Further inquiries can be directed to the corresponding author(s).

## Supplementary Materials

The following supporting information can be downloaded at: https://www.mdpi.com/article/10.3390/molecules30061215/s1, Table S1: Data on UV-Vis and Raman Spectra.
